# An In vitro Comparison of Apically Extruded Debris Using Reciproc, ProTaper Universal, Neolix and Hyflex in Curved Canals

**DOI:** 10.22037/iej.v12i3.13540

**Published:** 2017

**Authors:** Hossein Labbaf, Kiumars Nazari Moghadam, Shahriar Shahab, Mahshid Mohammadi Bassir, Mohammad Amin Fahimi

**Affiliations:** a *Department of Endodontics, Dental School, Shahed University, Tehran, Iran; *; b *Department of Oral and Maxillofacial Radiology, Dental School, Shahed University, Tehran, Iran; *; c * Department of Operative Dentistry, Dental School, Shahed University, Tehran, Iran; *; d *Dentist, Dental School, Shahed University, Tehran, Iran*

**Keywords:** Controlled Memory, Debris Extrusion, Reciprocating, Root Canal Preparation, Rotary Instrumentation

## Abstract

**Introduction:**

As a consequence of root canal preparation, dentinal chips, irrigants and pulp remnants are extruded into preradicular space. This phenomenon may lead to post endodontic flare-ups. The purpose of this study was to compare the amount of extruded debris with four endodontic NiTi engine-driven systems.

**Methods and Materials:**

Sixty mesiobuccal roots of maxillary molars with 15-30˚ curvature were divided randomly into four groups (*n*=15). Each group was instrumented up to apical size of 25 using Reciproc, ProTaper Universal, Neolix and Hyflex. Bidistilled water was used as irrigant and extruded debris was collected in pre-weighted Eppendorf tubes. Tubes were stored in incubator for drying the debris. Extruded debris were weighted in electronic microbalance with accuracy of 0.0001 g. The raw data was analyzed with one way analysis of variance (ANOVA) and Tukey’s HSD post hoc test. Level of significance was set at 0.05.

**Results:**

The debris extrusion with Reciproc files was significantly higher than the other groups (*P*<0.05). Hyflex significantly extruded less debris than other files (*P*<0.05). There was no significant difference between ProTaper Universal and Neolix regarding the amount of extruded debris (*P*=0.98).

**Conclusion:**

All systems extruded debris during the instrumentation. Reciproc system significantly extruded more debris. Caution should be taken when interpreting the results of this study and applying it to the real clinical situation.

## Introduction

As one of the most important steps for root canal treatment, chemomechanical preparation, may harbor negative consequences: extrusion of dentinal chips, pulp tissue remnants, microorganisms and irritants into the periradicular space which may lead to inflammation and delayed healing [1]. This postoperative complication known as flare-up, may cause pain and/or swelling and urgent dental visit [2]. Incidence of flare up is estimated to be 1.4-16% [3]. This is partly dependent on iatrogenic errors and host related factors [4].

Currently, no instrumentation technique can eliminate the extrusion of debris. In many previous studies, hand instrumentation technique showed more extruded debris than engine-driven instrumentation [5-7].

Reciproc (VDW, Munich, Germany), and ProTaper Universal (Dentsply Maillefer, Ballaigues, Switzerland) are well-known engine driven systems. ProTaper is a full sequence conventional rotary system with full rotational movement, while Reciproc is a single-file system with reciprocating (150˚ counter-clockwise then 30˚ clockwise rotation) motion, made of a special alloy named M-wire. M-Wire alloy has proved to be more flexible and increased resistance against cyclic fatigue [8, 9]. In addition, reciprocating motion, eliminates the engagement between instrument blades and dentinal walls. This may decrease the cyclic fatigue of the instrument [10]. Many studies showed that Reciproc extruded more debris than ProTaper Universal [11, 12]. However, De Deus *et al.* [13] and Silva *et al. *[14] showed different results.

Recently, Hyflex CM (Coltene-Whaledent, Allstetten, Switzerland) and Neoniti (Neolix, Châtres-la-Forêt, France) rotary instruments have been introduced which are made of a controlled memory (CM) NiTi wire [3]. These instruments are made by thermal treatment of the conventional NiTi wires. This structure controls the material memory and has excellent flexibility and resistance to cyclic fatigue [15]. Hyflex CM is available in various tapers and tip sizes. Neolix is available in C1 (25/0.12) for coronal flaring and A1 (25/0.08) for middle and apical regions. A Previous study showed that Hyflex CM is safe and efficient in cleaning and shaping of the root canal system [16]. However, cleaning ability and safety of the Neolix system is not quite known. 

This is necessary to perform more studies on Neolix for understanding properties of this newly introduced system. In addition Hyflex CM and Neolix are made of CM wires and need to be compared in amount of extruded debris. The aim of this study was to compare amount of extruded debris in ProTaper Universal and Reciproc systems with those in Hyflex CM and Neolix. The null hypothesis tested was that there is no significant difference in amount of extruded debris in four above mentioned systems.

## Materials and Methods

***Sample selection***

A total of 60 human mesiobuccal roots of first maxillary molars were collected. Inclusion criteria was mature apices, angle of curvature between 15˚ to 30˚ according to the Schneider’s method [17], radius of curvature less than 10 mm [18], minimum length of 19 mm [19] and non-calcified canals. These were confirmed by CBCT imaging of the teeth. Teeth with calcification, internal or external root resorption, severe curvatures, open apices and cracked roots were excluded. In addition, all specimens were controlled with CBCT for the mesiopalatal (MB2) canal and only mesiobuccal (MB) canal was prepared. Specimens were immersed in 0.5% sodium hypochlorite solution for 48 h for disinfection. Before instrumentation, soft tissue, pulp remnants and calculus were removed mechanically from root surface by a periodontal scaler.

***Root canal preparation***

The crown of the teeth were cut off 3 mm above the CEJ level with a corborundum disk so all samples had about 19 mm length. For ease of instrumentation, all roots except mesiobuccal root were separated. Mesiobuccal root canals with an initial apical size of #15 K-file (Mani, Tochigi, Japan) which was confirmed by radiography, were selected for this study. Initial instrument was inserted to the apical foramen and subtracted 1 mm to obtain the working length (WL). Apical patency was gained with a #10 hand K-file (Dentsply, Maillefer, Ballaigues, Switzerland).

The teeth were divided into 4 groups (*n*=15) and in each of these groups, the apical preparation was done up to a size 25 instrument according to the manufacturer’s specification. For a smooth and negotiable canal, glide path was created in all specimens. 

All of the instrumentations were operated by a low torque motor (VDW Silver, VDW, Munich, Germany) with 6:1 reduction handpiece. Each file, was used under its individual rotational speed and torque limit which was preprogrammed in the memory of the motor.

After using each instrument or 3 peckings of Reciproc files, the canals were irrigated by 2 mL of bidistilled water with a side vented 27-G needle (Ultradent, South Jordan, UT, USA). Each instrument was used for 4 canals and all of the instrumentations, were done by one operator. The instrumentation sequence was as follows: In Reciproc group; as recommended by the manufacturer, a R25 Reciproc file (25/0.08) was used in a slow in-and-out pecking motion. The flutes of the files were cleaned after 3 pecks. The instrument was reached to the WL. In ProTaper Universal group; the sequence of instrumentation was done as instructed by the manufacturer. SX at two thirds of WL or before initiating of the curve, S1 and S2 at 1 mm short of the working length, F1 (20/0.07) and F2 (25/0.08) at WL and once rotated freely, it was removed. All the instruments used under a brushing and gentle in-and-out motion. In Neolix group, brushing and gentle in-and-out motion used for instrumentation. These files were used as the manufacturer instruction with sequence of C1 (25/0.012) at coronal third of the canal and A1 (25/0.08) at WL. In Hyflex group gentle in-and-out motion was used during instrumentation. These files were used as the manufacturer instruction with the sequence of 25/0.008 at two third of WL and 25/0.06 at WL.

***Collection of extruded debris***

The experimental model which has been described by Myers and Montgomery was used to determine the amount of extruded debris [20]. In the stopper of an Eppendorf tube (Eppendorf India Limited, Chennai, India), a hole with the same diameter of the roots was prepared and a tooth was placed under pressure through this rubber stopper up to the cementoenamel junction. A 25-G needle is placed into the rubber stopper to equalized internal and external pressures of the tube. The unit including tooth and needle was fixed to the cover with cyanoacrylate. Then the apparatus (including stopper, needle and tooth) was attached to a vial with Putty (Coltene-Whaledent, Allstetten, Switzerland) silicon impression material for stabilization. Dark rubber dam was placed to obscure the tube from operator’s vision. Before instrumentation, empty tubes were weighted with electronic semi-micro balance (Sartorius AG, Gottingen, Germany) with accuracy of 10^-4 ^g.

After instrumentation, teeth were separated from the rubber stop and the debris adhering to the toot surface, were collected by washing the tooth with 1 mL bidistilled water. Tubes were stored in incubator for 5 days at 70˚C to evaporate the moisture before weighting the dried and net debris. Three consecutive weights were obtained from each tube and the mean was calculated. Evaluation of the amount of extruded debris was performed by another examiner which was blind to the details of the experiments. The weight of dried debris was obtained by subtracting the weight of tube after instrumentation from weight of tube before instrumentation.

The amount of extruded debris were analyzed statistically using the one way analysis of variance (ANOVA) and Tukey’s post hoc test at a significance level of 0.05.

## Results

Extrusion of debris were observed in all samples. There were significant differences between the groups (*P*<0.05) (Table 1).

Reciproc system produced significantly more extruded debris than the other groups (*P*<0.05). There was no significant difference between Neolix and ProTaper Universal (*P*>0.05). Hyflex CM produced significantly less extruded debris than the other groups (*P*<0.05).

## Discussion

The results of this study revealed that extrusion of debris occurred in all groups independent of type of instrument that used. Different types of instruments extruded different amounts of debris, so the null hypothesis of this study was rejected.

In most of the previous studies, single and straight root canals were used due to ease of instrumentation and predictable results [11, 12, 21, 22]. In clinical practice, most of the endodontic treatments were done on root canals with mild to moderate curvature. So, molars were enrolled into this study for closer *in vivo *circumstances. Only one study found using maxillary molars [23], and most of the previous studies were done using mandibular molars [13, 24]. In addition mesiobuccal canal of maxillary molars, have a complex anatomy which can be challenging in clinical practice. However this study wasn’t designed to compare straight canals and curved canals in amount of extruded debris. 

The type of irrigant, may have effect on the amount of extruded debris [25]. However, sodium crystallization phenomenon, can affect the results of the study if sodium hypochlorite is used [26]. Therefore, bidistilled water was used as irrigation solution.

Neither method nor system can avoid extrusion of debris [6, 10-13, 21, 22, 24, 27-29]. In accordance to the results of this study, many previous studies showed more extruded debris in Reciproc systems than full sequence and single rotary instruments [11, 12]. These results may be related to the s-shaped cross sectional design and sharp cutting edges of Reciproc file [12, 30]. Therefore, Reciproc system has superior cutting efficiency [31]. As a result, more debris and dentinal chips were produced. In addition, reciprocating motion, seem to increase the extrusion of debris beyond the apical foramen [11, 12]. On the other hand, using orifice shapers such as SX in ProTaper Universal system, can lead to a better crown-down concept in multi-file systems and less extruded debris.

There was no significant difference between ProTaper Universal and Neolix (*P*=0.98). Neolix A1 instruments have non-homothetic rectangular cross section with abrasive surface [32], while the ProTaper Universal F2 has a convex triangular cross section [12]. As a result, Neolix may have a superior cutting efficiency than ProTaper. On the other hand, a study concluded that more extruded debris are associated with full sequence rotary files such as ProTaper Universal because of several times of irrigation and insertion of instrument in canal [13]. However this study didn’t determine the relationship between the number of instruments used and extrusion of debris. 

**Table 1:** Amount of apically extruded debris produced by different systems (in gram) (Different superscript letters=significant difference between groups

**Group (N=15) **	**Mean (SD)**	**Min**	**Max**
**Reciproc **	0.00276 (0.000437)^a^	0.0019	0.0036
**ProTaper Universal **	0.00232 (0.000410)^b^	0.0019	0.0034
**Neolix **	0.00236 (0.000394) ^b^	0.0017	0.0033
**Hyflex **	0.00171 (0.000322)^c^	0.0012	0.0022

Hyflex system significantly extruded less debris than the other groups. In one study, Hyflex extruded less debris than ProTaper Universal [21], which is in accordance to our study. Unwinding the spirals of Hyflex, occurs during of the instrumentation of all specimens. This phenomenon may lead to decrease in the cutting ability and cleaning efficiency of instrument. As a result, production of dentinal chips and debris were decreased and less extrusion of debris happened in the samples. Final instrument that was used in Hyflex group had a 0.06 taper while in Neolix it had 0.08 taper. This may explain the more amount of extruded debris in Neolix group.

The experimental model described by Mayers and Montgomery [20] was used to collect the extruded debris. This apparatus had an important disadvantage that the apices were suspended in the air and didn’t mimic the vital periapical tissues and their back pressures which was discussed in previous studies [11-13, 24]. Therefore, the results of this study can’t be completely adopted to the clinical situations and transferring them to the clinical situations should be done under caution. Some authors has been proposed using of floral foam in order to stimulate back pressure of periapical tissues [33, 34]. This method has the disadvantage of absorption of the extruded debris and irrigants. Hence in our study, the resistance of periapical tissues wasn’t simulated. 

## Conclusion

All systems were associated with extrusion of debris. Reciproc files significantly extruded more debris than other systems. Hyflex files significantly extruded less debris than the other systems. There was no significant differences between Neolix and ProTaper Universal in amount of extruded debris. We can conclude that the concept of crown-down technique is followed better in multiple sequence systems than single-file systems such as Reciproc system.
